# Pancreatic Cancer Patient Survival Correlates with DNA Methylation of Pancreas Development Genes

**DOI:** 10.1371/journal.pone.0128814

**Published:** 2015-06-03

**Authors:** Michael J. Thompson, Liudmilla Rubbi, David W. Dawson, Timothy R. Donahue, Matteo Pellegrini

**Affiliations:** 1 Department of Molecular, Cell, and Developmental Biology, University of California Los Angeles, Los Angeles, California, 90095, United States of America; 2 Department of Pathology and Laboratory Medicine, University of California Los Angeles, Los Angeles, California, 90095, United States of America; 3 Department of Surgery, University of California Los Angeles, Los Angeles, California, 90095, United States of America; 4 Department of Molecular and Medical Pharmacology, University of California Los Angeles, Los Angeles, California, 90095, United States of America; 5 Jonsson Comprehensive Cancer Center, University of California Los Angeles, Los Angeles, California, 90095, United States of America; New York University School of Medicine, UNITED STATES

## Abstract

DNA methylation is an epigenetic mark associated with regulation of transcription and genome structure. These markers have been investigated in a variety of cancer settings for their utility in differentiating normal tissue from tumor tissue. Here, we examine the direct correlation between DNA methylation and patient survival. We find that changes in the DNA methylation of key pancreatic developmental genes are strongly associated with patient survival.

## Introduction

Pancreatic cancer is the fourth leading cause of cancer-related deaths in the United States [1]. The 5-year survival rate of 6% for patients with pancreatic ductal adenocarcinomas (PDAC) is the lowest for any solid cancer. Early detection is considered essential in order to improve patient survival as most patients present with advanced, non-operable disease [2, 3]. Recent advances in technologies for genome-wide measurements raise hope for the identification of such early biomarkers, as well as new therapeutic targets.

Multiple deep sequencing efforts have identified large numbers of highly heterogeneous mutations (~63 on average) with four mutations occurring at high frequency (KRAS, CDKN1A/P16, TP53 and SMAD4) in PDAC [4, 5] These key mutations have been shown to mechanistically drive tumorigenesis in animal models with SMAD4 associating with poor prognosis [6]. Gene expression analysis of primary tumors has enabled construction of multi-gene profiles that can predict metastatic disease and shorter patient survival in independent datasets but these have yet to be used in clinical decision making [7, 8]. By combining multiple sets of clinical data, three molecular subtypes of PDAC that predict survival and response to therapy in experimental models have been developed [9]. Recently, 171 genes with predictive biomarker potential were identified by combining mRNA expression, DNA copy number variation and miRNA levels [10].

However, to date less is known about changes in DNA methylation across pancreatic cancer subtypes. DNA methylation has gained much recent interest for its role in cancer biology. Aberrant patterns of DNA methylation are known to be associated with carcinogenesis and to affect the regulation of genome stability and gene transcription [11]. Genome-wide studies of CpG islands have uncovered thousands of loci where differential methylation can segregate pancreatic tumor tissue from normal tissue [12, 13]. Despite this progress, the use of changes in DNA methylation for predicting pancreatic patient survival remains unexplored.

Here we examine the direct correlation between PDAC patient survival time and methylation of individual CpG sites obtained from reduced-representation bisulfite sequencing (RRBS). Numerous statistically significant changes in methylation correlated directly with patient survival. We observed a strong enrichment of these sites among genes involved in cell-fate determination in the pancreas. In contrast to sequencing efforts that identified cellular signaling pathways, these results suggest that cellular identity, as dictated by the tumor’s epigenome, may be a critical component of clinically aggressive PDAC. Finally, we have further validated the ability of a few example sites to segregate patients based on survival times, which suggests the possibility of developing clinical biomarkers based on more extensive methylation analysis.

## Results

### Clinicopathologic characteristics of samples

Table 1 provides a summary of statistics for the patients used in this work. Individual patient data can be found in S1 Table. All patients had early-stage PDAC and received adjuvant chemotherapy. At the time of analysis, 9 patients had recurrent disease (median Disease-Free Survival (DFS) of 15.0 months), while 9 patients had died of disease (median Disease-Specific Survival (DSS) of 25.0 months). DFS refers to the interval between treatment or removal of the tumor and recurrence of the disease, while DSS refers to the interval between original diagnosis and patient death where cause of death was the disease. For this work we have used DSS exclusively. At the time of this analysis 9 patients were deceased and 2 were still living. These clinicopathologic characteristics and survival outcomes are similar to other published cohorts of PDAC.

**Table 1:** Clinical, histopathologic, and survival information for the 11 patients used in this study.

Factor	Subcategory	*N* (%)
Total samples		11
Age, y	Median (range)	65.0 (49–81)
	< 65	5 (45%)
	> = 65	6 (55%)
Survival, months	Median (range)	25.0 (9.3–70.2)
DFS, months	Median (range)	15.0 (5.4–70.2)
Tumor cellularity	Median (range)	75 (60–90)
Tumor diameter, cm	Median (range)	2.2
	< 2.5	6 (55%)
	> = 2.5	5 (45%)
T stage	2	5 (45%)
	3	3 (27%)
	4	3 (28%)
Tumor differentiation	Well	0 (0%)
	Moderate	5 (45%)
	Poor	6 (45%)
Lymph nodes	Positive	8 (73%)
	Negative	3 (27%)

### Reduced-representation bisulfite sequencing

Mapping of all quality reads to the reference human genome (hg19) yielded ~5 million CpG sites where a confident methylation frequency could be estimated in at least one of the 16 samples. Application of the data quality filters explained above reduced this to a final number of 251,566 methylation sites used in all subsequent analysis.

### Principal Component Analysis Separates Tumor from Non-tumor

**Fig 1:**
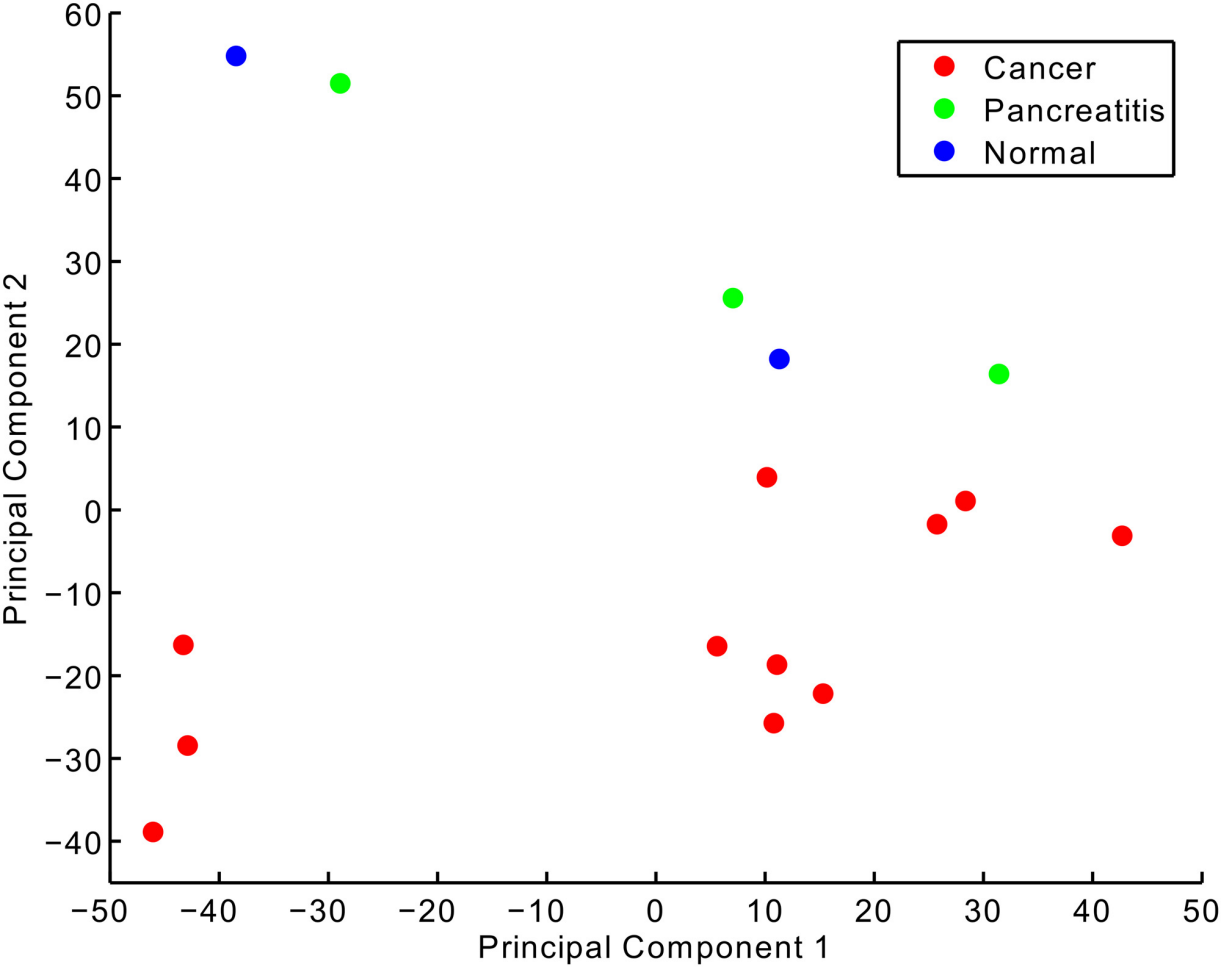
Principal Component Analysis of methylation profiles for eleven PDAC tumor samples, three pancreatitis samples, and two normal samples.

Principal component analysis (PCA) of the methylation profiles for the 16 samples was performed. Fig 1 shows a projection of the samples onto the first two principal axes. Along the first principle component there is separation of the tumor samples. Along the second principle axis there is a clear separation between the tumor samples and the normal and pancreatitis samples. The first component explains 24% of variance in the methylation data while the second component explains an additional 21%.

To gain insight into the biology underlying the separation of samples along the two principle components, we selected the 500 largest magnitude coefficients for both positive and negative directions along each axis. As these coefficients correspond to CpG sites, we submitted them to the Genomic Regions Enrichment of Annotations Tool (GREAT) server for annotation [14]. For the first principle axis (see S2 Table) the strongest negative contributions come from genes involved in pancreatic development (e.g. ONECUT1, PDX1, SOX9, FOXA2,…). The strongest positive contributions, however, do not come from a set of genes with as coherent a functional annotation as the negatives except with regard to insulin trafficking and secretion; BACE2 and PIM-3, which is also known to promote human pancreatic cancer growth [15].

For the second principle axis (see S3 Table) the strongest negative contributions come from developmental genes for of a large number of tissue types and organ systems. The strongest positive contributions come from transmembrane receptor protein kinases. The most notable contribution comes from MST1R which has been implicated in KRAS oncogene activation in PDAC [16].

Given the separation among tumor samples based on methylation of genes important for development, we moved to examine whether there might be a correlation between methylation of these genes and the disease-specific survival of the patients.

### Definition of *Survival+* and *Survival-* terms for methylation sites

We observed that methylation sites with positive or negative Cox regression scores formed two groups with distinct properties, as discussed below. To simplify the conceptualization and referencing of these two groups, we defined them as “*survival-”*or “*survival+*”, both significantly correlated with increased methylation. For *survival-*, increased methylation was associated with shorter survival times. Conversely, for *survival+* increased methylation was associated with longer survival times. Based on our filtering criteria and applying a p ≤ 0.05 threshold for significance, we obtained a set of 17,251 *survival+* sites and 3256 *survival-* sites.

### Individual CpG site methylation correlates with patient survival

We tested whether other clinical variables known to affect DNA methylation might account for the correlations we observe. We performed the same Cox regression with censoring for each site’s methylation against both patient age and tumor content (cellularity). We then took the Cox scores based on survival and those based on each of the other variables and calculated the Pearson correlation of the two coefficients among all the sites in each of the *survival+* and *survival-* sites. For tumor quality, the correlation was 0.06 and for age, it was 0.08. Use of just the sites of interest to this study (those with p-value < 0.05 in the survival and methylation comparison) yielded correlations of 0.16 for age and -0.12 for tumor quality. Moreover the numbers of sites where correlation was statistically significant for both methylation and age or methylation and tumor content were quite small (n ~ 10). We therefore conclude that correlations between methylation levels and survival times are not significantly affected by patient age or tumor content.

### Functional analysis reveals keys genes in pancreatic cell fate commitment

Using the *survival+* and *survival-* methylation sites individually and the entire ~250K methylation sites as background, we used the GREAT tool to search for significant associations with various functional categories and pathways [17]. A subset of the significant associations found by these queries are listed in Table 2 for the *survival-* sites and Table 3 for the *survival+* sites.

**Table 2:** Significant function and pathway associations for *survival-* sites.

	FDR Q-value	Enrichment	Number of Genes
**GO Biological Process**			
regulation of transcription, DNA-dependent	8.13E-63	1.7	347
cell differentiation	7.36E-54	1.7	300
positive regulation of transcription from RNA polymerase II promoter	7.03E-43	2.2	96
neuron differentiation	1.78E-38	2	135
cell migration	2.31E-29	2.1	71
cell projection morphogenesis	3.08E-29	2.1	86
mesenchymal cell development	9.08E-28	3.5	23
neural crest cell migration	1.54E-23	4.8	12
axonogenesis	1.51E-21	1.9	81
Wnt receptor signaling pathway, calcium modulating pathway	3.92E-18	8.9	6
pancreas development	5.93E-17	2.6	16
chromatin assembly	5.52E-16	4.3	10
axon guidance	1.65E-15	1.9	60
type B pancreatic cell development	4.08E-15	8.7	2
negative regulation of synapse assembly	2.17E-12	10.2	2
neuron fate commitment	2.24E-12	2.4	22
negative regulation of transforming growth factor beta receptor signaling pathway	2.98E-12	3.4	6
epithelial to mesenchymal transition	3.11E-11	3.2	11
regulation of programmed cell death	5.23E-11	1.5	104
type B pancreatic cell differentiation	1.45E-10	5.5	2
regulation of angiogenesis	7.93E-09	2.5	15
neural precursor cell proliferation	8.86E-09	2.9	10
regulation of cell migration involved in sprouting angiogenesis	1.26E-07	7.6	2
**Pathway Commons**			
Regulation of gene expression in early pancreatic precursor cells	2.48E-12	6.2	1
FOXM1 transcription factor network	3.45E-10	4.5	3
Regulation of beta-cell development	5.94E-10	2.7	9
Synthesis, Secretion, and Inactivation of Glucose-dependent Insulinotropic Polypeptide (GIP)	1.32E-08	5.7	2
Neurofascin interactions	3.33E-08	9.2	3
Signaling events mediated by HDAC Class III	9.27E-07	4.3	5
Class B/2 (Secretin family receptors)	9.39E-07	2.9	8
Biosynthesis of the N-glycan precursor (dolichol lipid-linked oligosaccharide, LLO) and transfer to a nascent protein	1.05E-06	5.2	3
Noncanonical Wnt signaling pathway	1.39E-06	2	21
Post-chaperonin tubulin folding pathway	1.35E-06	5.8	2
Syndecan-4-mediated signaling events	1.16E-05	1.9	23
Incretin Synthesis, Secretion, and Inactivation	2.81E-04	2.8	5
Interleukin-1 signaling	3.57E-04	3.2	4
Serotonin receptors	3.62E-04	6.3	3
Validated targets of C-MYC transcriptional repression	6.05E-04	2.5	8
**Transcription Factor Targets**			
Targets of Oct4, identified by ChIP-chip in embryonic stem cells	2.15E-15	2.4	26
Targets of Sox2, identified by ChIP-chip in embryonic stem cells	1.47E-15	2	45
Targets of Nanog, identified by ChIP-chip in embryonic stem cells	1.09E-10	1.7	57
Targets of CREB, identified by ChIP-chip in HEK293T cells in three different time points after forskolin stimulation	3.40E-05	1.3	100

**Table 3:** Significant function and pathway associations for *survival+* sites.

	FDR Q-value	Enrichment	Number of Genes
**GO Biological Process**			
detection of chemical stimulus involved in sensory perception	4.2E-04	1.7	54
nucleotide-binding oligomerization domain containing 2 signaling pathway	5.1E-04	6.2	3
sensory perception of smell	9.8E-04	1.5	59
NF-kappaB binding	5.8E-04	2.3	12
olfactory receptor activity	6.1E-04	1.7	46
**PANTHER Pathway**			
p53 pathway feedback loops 2	7.4E-04	1.8	24

For the *survival-* set of methylation sites, we observed a number of significant associations with developmental processes and pathways specific to the pancreas, including key genes involved in transcriptional regulation determining cellular differentiation in the pancreas. Links were also noted between genes and processes of neural cell differentiation, a finding noted previously in a both an exome sequencing effort [5] and a DNA methylation study of PDAC [13].

**Fig 2:**
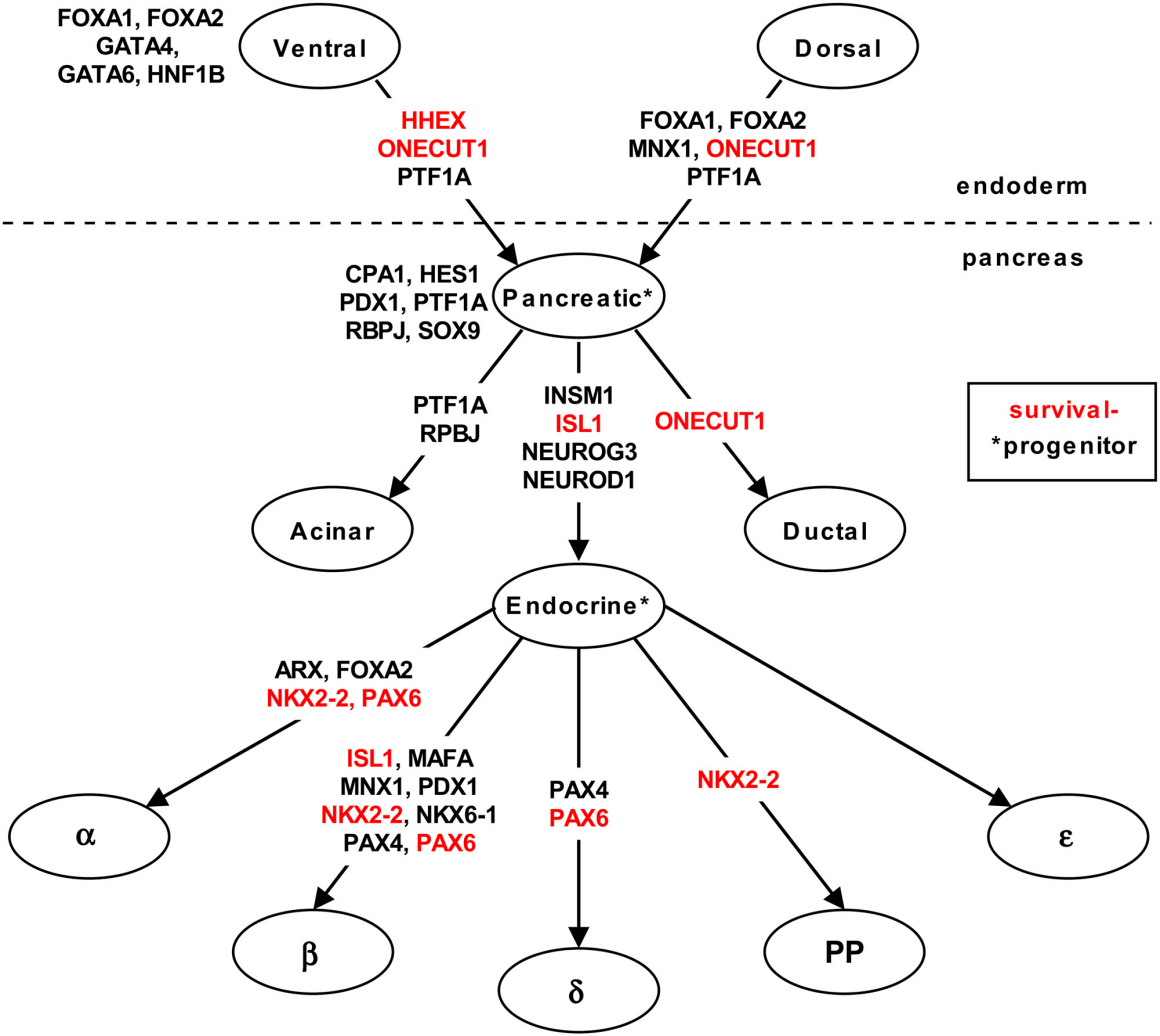
Schematic of cell lineage relationships in the pancreas. Genes that are well-established as playing key roles in cell fate are listed by name. Names highlighted in red are genes enriched for *survival-* methylation sites.

Fig 2 is a schematic of cell lineages in the pancreas adapted from Zaret, *et al*. [18]. Genes highlighted in red were enriched in the *survival-* set, including HHEX, ONECUT1, ISL1, NKX2-2, and PAX6. HHEX, a homeobox gene, is critical for pancreatic development and ONECUT1 is necessary for the timely expression of PDX1 and NEUROG3 in both dorsal and pancreatic endoderm [19, 20]. NKX2-2 is required for pancreatic beta cell development [21], ISL1 is required for islet cell development [22], and, PAX6 is required for the differentiation of alpha cells [22].

It has been previously reported that the hypermethylation of ISL1 correlates with decreased gene expression and aggressive progression of invasive bladder cancer [23]. The methylation of NKX2-2 was identified as part of a signature for glioblastoma multiforme [24]. Finally, the hypermethylation of PAX6 was observed to correlate with poor clinical outcome in gastric cancer [25, 26], development of non-small cell lung cancer [27] and transcriptional deactivation in invasive ductal breast carcinoma [28, 29].

### *Survival-* and *survival+* have distinct positional propensities

**Fig 3:**
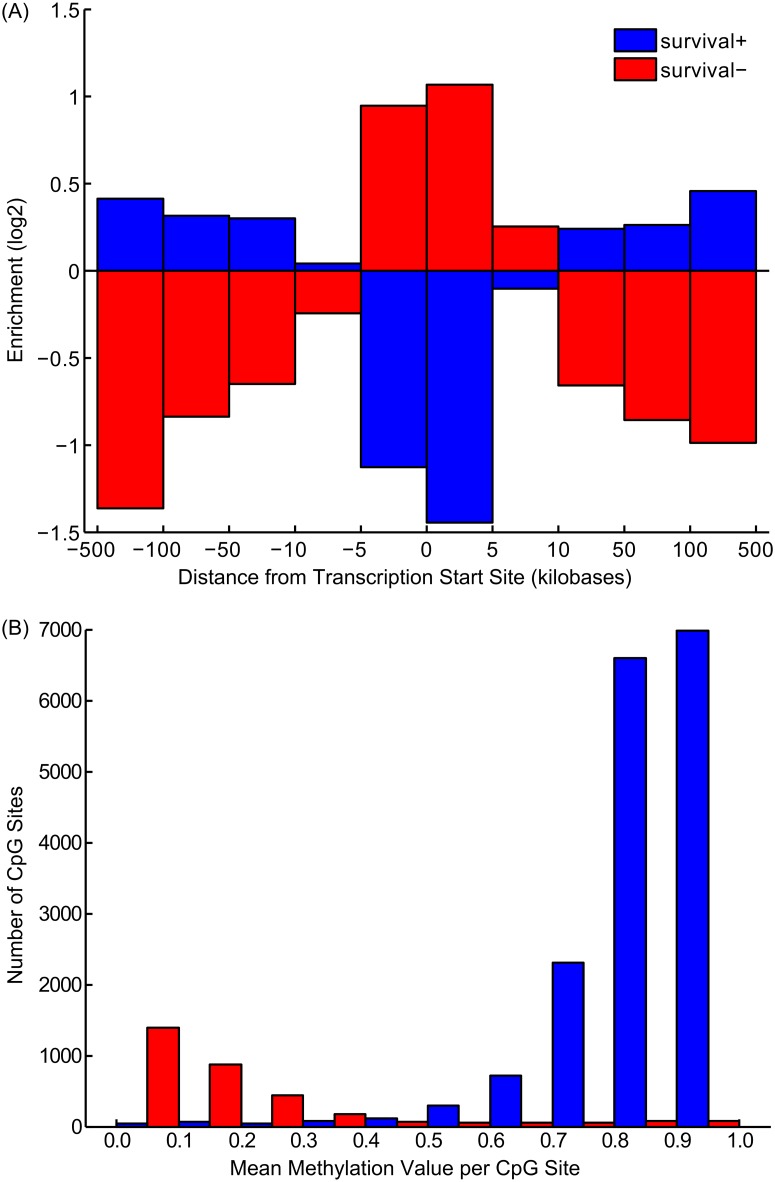
(A) Enrichment (log_-_odds ratio) of *survival+* and *survival-* sites in different distance bins relative to the transcriptional start sites (TSS) of their nearest genes. (B) Histogram of average methylation scores for CpG sites categorized as *survival-* and *survival+*.

Fig 3A plots the log-odds ratios for *survival-* and *survival+* sites at various distances from the transcription start sites of their nearest genes. The *survival-* sites, which have the clearest annotation results, were substantially enriched in the promoter regions of genes. In contrast, the *survival+* methylation sites, which, as a group, have less coherent association with particular functional annotations were distributed more broadly and distal to the genes’ transcriptional start sites. Similarly, the two categories of methylation sites have distinct statistical properties. Fig 3B is a histogram of average methylation values for methylation sites of both categories. Survival- and survival+ sites appear to have nearly separate and distinct distributions of methylation values. While survival- sites tend to have low methylation that increases with shortened survival times, the survival+ category is overwhelmingly comprised of CpG sites that are hyper-methylated. We note that these two distributions reflect the background distribution of all sites (see S1 Fig). The abundance of survival+ sites relative to survival- sites reflects the larger background distribution of hypermethylated CpGs. As hypomethylated regions of the genomes are typically associated with transcriptional start sites, it is likely that the survival- sites are more directly related to transcriptional changes than the survival+ sites, which are often in distal intergenic regions, and have a less clear relationship to transcriptional regulation.

### Toward Biomarker Development

**Fig 4:**
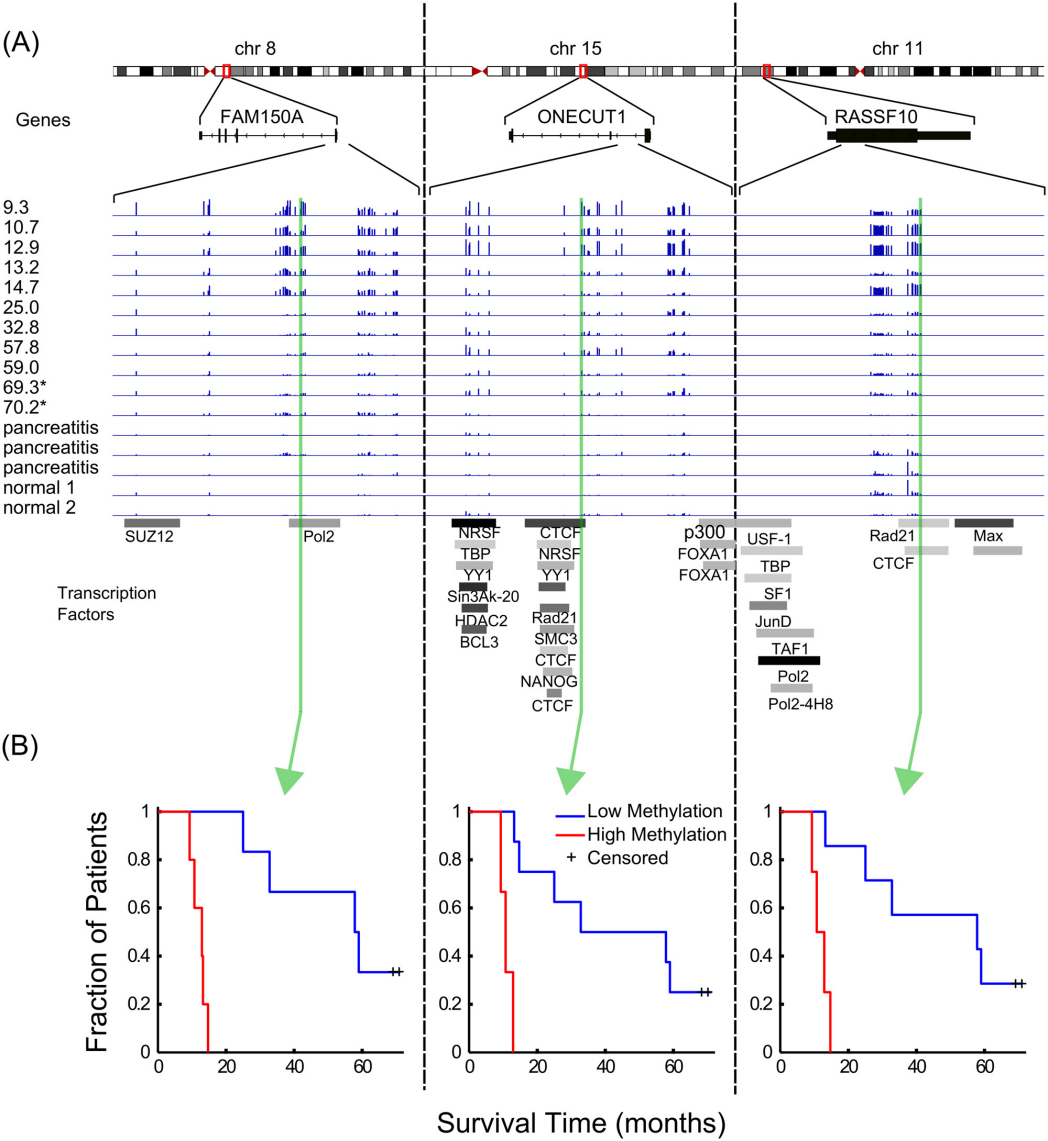
Genome browser visualization of DNA methylation, patient survival, and gene structure. (A) Three genes where increased methylation in the promoter region strongly correlates with decreased patient survival. Also shown are tracks corresponding to transcription factor binding evidence. (B) Kaplan-Meier curves for one site from each gene occurring within a transcription factor binding site.

Specific examples of the correlation between methylation and patient survival are presented in Fig 4. Fig 4A displays three genes (FAM150A, ONECUT1, and RASSF10) that were locally enriched for *survival-* sites. The panel shows the chromosomal location, the structure, and a magnified view of the DNA methylation tracks for the 11 PDAC patients with their survival time in months listed on the left of the track as well as tracks for 2 pancreatitis and 3 normal samples. The lowest portion of the Fig 4A panel depicts tracks for transcription factor binding regions obtained from the ENCODE project [30]. Fig 4B depicts Kaplan-Meier curves for three specific sites—each taken from the gene immediately above it. A complimentary set of examples using *survival+* sites is provided in S2 Fig.

### Comparison to known PDAC-associated genes

Employing a gene-centric approach, we counted the number of significant methylation sites (*survival+* or *survival-*) occurring in a given gene plus a 5 kilobase extension upstream of the transcription start site. We then sorted these two lists and examined the top 10 entries in each. The complete lists of genes and the number of significant methylation sites for each are given in S4 Table.

Additionally, we looked at overlap between our gene sets and sets of genes with mutations uncovered in two extensive sequencing studies of pancreatic cancers [4, 5]. For our gene lists, we took only those that had 10 or more significant methylation sites (to reduce the false discovery rate of any single site). This gave us 32 genes for the *survival-* set and 54 genes for the *survival+* set. Only one (COL5A1) appeared in both a list of mutated genes [4] and the *survival+* list, while no overlap was noted with the *survival-* set. This lack of overlap between genes enriched for survival- and survival+ methylation sites and genes associated with pancreatic cancer mutants is explained by two factors. The first is that we applied stringent criteria for genes to be considered in order to minimize false discovery, and therefore we have not necessarily captured all epigenetic changes. The second, and probably more important observation, is that PDAC-associated mutant genes are not enriched for pancreatic developmental genes, but rather for signaling pathways. Thus mutations to these signaling pathways likely confer survival benefits for the tumors, while developmental changes that confer survival benefits are more efficiently modulated though epigenetic rather than genetic changes.

## Discussion

Genomic alterations and gene expression changes associated with pancreatic cancer have been studied intensively, and a number of critical genes have been identified [4, 31, 32]. Quantitative integration of multiple data-sources for genomic alterations, gene expression, and epigenetic regulation with patient survival has aided in characterizing the relationships between the PI3K/AKT and SRC pathways [10] and PDAC progression. In contrast to previous studies, we have adopted the *survival-*based approach with a focus on potential epigenetic regulation via DNA methylation.

We found that the methylation of CpG sites correlated with patient survival in ~20,000 instances out of ~250,000 measured. These sites fell into two categories we called *survival-* and *survival+* representing the relationship of increased methylation to survival. We also found that these two types of sites had distinct positional propensities relative to the transcriptional start sites of genes. While the *survival-* sites clustered near the TSS—corresponding to hypermethylation of promoter regions, the *survival+* sites were more broadly distributed intragenically. As a result of this partitioning, and the generally more fully understood and characterized promoter regions of genes, the *survival-* sites yielded clearer recovery of functional annotation and genes in the aggregate.

While the vast majority of DNA methylation studies have focused on the methylation status of promoters and CpG islands, there is growing evidence that intronic methylation and/or methylation sites that are not part of CpG islands also play a role in modulating gene expression [33, 34]. It is thought that these methylation of these intra-genic sites affects the role of enhancers of transcription [35–37]. Our study lends credence to this as the bulk of significant methylation changes we observed were in the *survival+* category (not proximal to the TSS).

The gene FAM150A had the greatest number of *survival-* sites. While not well-characterized in the literature, in a recent study of renal cell carcinomas, it was found to be hypermethylated in the cluster of patients with aggressive cancer and poor survival [38, 39]. The role of ONECUT1 (HNF6) in pancreatic development is well established. In particular, it is thought to act as a phenotypic switch in acinar-to-ductal metaplasia that can lead to pancreatic intraepithelial neoplasia and subsequent PDAC [40]. Studies in mouse indicate ONECUT1 has tumor suppressor activity [41]. Another well-known tumor suppressor in our top 10 list is RASSF10. Recent studies have identified it as epigenetically silenced via promoter hypermethylation in a number of cancers [42–52]. RNF207 was also recently identified as a tumor suppressor in neuroblastomas [53]. PCDH9 is frequently lost in hepatocellular carcinomas, its down-regulation is associated with tumor cell migration, and PCDH9-negative tumors correlate with significantly shorter survival times for glioma patients [54, 55]. Likewise, the loss of one allele of BRF1 is associated with a variety of different tumors [56]. As a subunit of TFIIB, the deregulation of this gene likely contributes to the deregulation of RNA pol III transcription widely observed in cancer cells [57].

Of the top 10 *survival-* genes, only three (MAP6, RIN3, and HIST1H2BI) lacked clear-cut evidence of an oncogenic or tumor suppressor role in the literature. RIN3 is a negative regulator of mast cell response to stem cell factor [58], and may play a suppressive role in tumor invasiveness. HIST1H2BI does not appear to have a known role in cancer aside from a general chromatin structural role.

The remaining gene in our top 10 list encodes an miRNA (MIR96) known to suppress KRAS and function as a tumor suppressor in pancreatic cancer [59]. As a result, we also examined other miRNAs appearing in our *survival-* gene list. Though not in the top 10, we found two more miRNA genes with more than 5 significant methylation sites. MIR130B is down-regulated in pancreatic cancer tissues and its expression is a useful prognostic for pancreatic cancer patients [60]. MIR196A1 has also been identified as having aberrant expression associated with abnormal apoptosis, invasion and proliferation of pancreatic cancer cells [61].

Examining the 10 genes with the most numerous *survival+* sites yielded the following findings. PTPRN2 is a well-known member of the major auto-antigens in insulin-dependent diabetes mellitus. It has also been identified as a hypermethylated biomarker in squamous cell lung cancer [62]. CDH4 is aberrantly methylated in its promoter region in gastric and colorectal cancer and may act as an epigenetically silenced tumor suppressor in nasopharyngeal carcinoma [63, 64]. MAD1L1 is a well-studied cell cycle checkpoint gene with mutations implicated in multiple types of cancer [65]. CBFA2T3 is found in pediatric acute myeloid leukemia and is involved in chromosomal translocations and fusion protein products (CBFA2T3-GLIS2) [66]. Aberrant methylation of CBFA2T3 promoter has been found in breast cancer tissue compared to normal [67]. CBFA2T3 is also found in IGH chromosomal translocations in pediatric B-cell lymphoma [68]. The collagen-remodeling gene, COL5A1, was used in a 10-gene expression signature associated with poor patient survival in high-grade serous ovarian cancer and its expression appears to promote metastasis [69]. CAMTA1 is a tumor suppressor candidate found to inhibit growth in neuroblastomas and appears to play a role in the development of glioma stem cells [53, 70–72]. The H3K9me1 methyltransferase PRDM16 helps protect genomic integrity [73]. Translocations of this gene are found in acute myeloid leukemia and myelodysplastic syndrome and correlated with poor patient survival [74]. SHANK2 has been suggested as a novel oncogene as its over-expression in esophageal squamous cell carcinoma is associated with cell proliferation and protection against cell death [75].

For two of the top 10 genes there was little literature annotation. High levels of the RBFOX4 protein have been found in supratentorial ependymomas.[76], while there is essentially no link in the literature to a cancer association for TRAPPC9.

As we found interesting miRNAs in our set of *survival-* genes, we looked for them in the *survival+* set, as well. Despite the larger numbers of *survival+* sites relative to survival site, there were no miRNA genes associated with more than 5 of this class of sites.

Following a closer inspection of individual genes associated with multiple occurrences of *survival+* and *survival-* sites, we found that nearly all had been identified via other studies with other types of data as playing a role in cancer, even if they have not been investigated specifically in relation to PDAC. Our findings are consistent with previous studies reporting correlations between promoter hypermethylation or transcription inactivation for several key developmental regulatory genes or genes with tumor suppressive capacity in other cancers. The pathways that are enriched for *survival-*related epigenetic changes correspond to some of the pathways previously identified in a broad sense. The appearance of 3 miRNA genes in our *survival-* set, all of which have been identified as key transcription regulators in PDAC, is of particulate note and gives us greater confidence in the approach we have taken.

Finally, as a preliminary test of the potential predictive utility of DNA methylation for pancreatic cancer patient survival, we tested 3 *survival-* sites occurring in 3 genes with multiple *survival-* sites at locations for which there is evidence of protein binding. The methylation values at each of these sites could dichotomize the patients into short survival and longer survival with statistical significance.

The results of this approach show clearly that DNA methylation changes of key cell fate determining genes is strongly associated with PDAC progression. This study should motivate collection of more sample data to confirm our results as well as to develop biomarkers based on DNA methylation changes associated with these key genes.

## Methods and Materials

### Patients and samples

All work was conducted with the approval of the University of California, Los Angeles (UCLA) Institutional Review Board with written consent from all patients. Samples from PDAC tumors and nonmalignant pancreas were snap frozen at the time of surgery. Tumor content was assessed on hematoxylin and eosin sections by a practicing gastrointestinal pathologist (DWD). All clinicopathologic and survival information for patients was extracted from a prospectively maintained UCLA surgical database of pancreatic patients. Overall survival was determined by searching the Social Security Death Index and survival intervals were calculated from date of surgery to date of confirmed death or last patient contact.

For this study, a total of 16 samples were used, including 11 PDAC tumors, 2 normal samples and 3 chronic pancreatitis samples. Clinical details for these samples are given in S1 Table.

### Reduced representation bisulfite sequencing

Genomic DNA from all samples was extracted to create libraries for reduced representation bisulfite sequencing (RRBS) following a standard protocol [77]. Digests were performed with the methylation-insensitive restriction enzyme, MspI, and fragments ranging from 50 to 300 bases were selected to enrich for CpG rich regions. After digestion, samples were ligated with Illumina adaptors, size selected, denatured and treated with sodium bisulfite to reveal their methylation state. Libraries were sequenced using Illumina Hiseq 2000 sequencers.

Sequencing reads were mapped to the human reference genome (hg19) using the BS Seeker2 set of computational tools [78]. As bisulfite treatment converts unmethylated cytosines to thymines, we computed the methylation level at each genomics CpG site as the fraction of reads uniquely mapped to that site containing a cytosine.

### Data Filtering

We filtered the set of methylation sites for subsequent analysis using two statistical criteria intended to select biologically relevant sites and improve the quality of downstream calculations. First, as the methylation state at a given site is a binomial frequency estimate, we computed confidence intervals using the Clopper-Pearson method [79]. We included only sites where methylation frequencies for each of the 16 samples had 95% confidence intervals less than 0.25. Second, we computed the standard deviation across the 16 samples and culled sites with a standard deviation < = 0.05 for further analysis.

### Statistical Analysis

Principal Component Analysis [80] was performed on methylation vectors comprised of all sites that passed the filters described above. Cox regression [81] with censoring was used to calculate a regression coefficient and associated p-value at each site individually to determine the sign and strength of relationship with the survival data. Likewise, for some sites of interest presented in Results and Discussion, we computed Kaplan-Meier [82] curves and log-rank p-values by dichotomizing the samples based on the average methylation value at each site.

### Function and Pathway Analysis

To investigate the functional implications of methylation sites with strong correlation to survival, we utilized the Genomic Regions Enrichment of Annotations Tool (GREAT) [17]. This tool aggregates multiple databases of biomolecular annotation and computes statistical associations between these annotations and a given input set of genomic regions. For our input, we used the subset of methylation sites with p-values less than 0.05. For the background, we used all sites that passed the two quality filters. For the GREAT input parameters we chose the single nearest gene option and a maximal extension of 100 kilobases from the transcription start site of each gene.

Given the large number of CpG sites examined in this study, we attempted to apply standard multiple-testing corrections [83] to these p-values. As a result of the under-representation of low p-values in our data compared to uniform random expectation, the false discovery rates we obtained was likely inflated. However, as shown in Results and Discussion, using uncorrected p-values for selecting loci in an aggregate manner (per gene or genomic region) provided convincing recovery of functional annotation and genes previously highlighted in the literature, suggesting our approach is robust. Finally, we compared the set of genes most strongly enriched with significant methylation sites to the lists of genes found to be significantly mutated in extensive exome sequencing project [4, 5].

## Supporting Information

S1 Fig Background Distribution of Per Site Average Methylation Values. (TIFF)

S2 Fig Genome browser visualization of Example Genes with Survival+ Sites. (TIFF)

S1 Table Clinicopathological characteristics of patient samples. Clinical variables collected for pancreatic adenocarcinoma patients in this study.
(XLSX)

S2 Table Functional Basis of Principle Component 1. (XLSX)

S3 Table Functional Basis of Principle Component 2. (XLSX)

S4 Table Genes with significant DNA Methylation sites. Two lists of genes rank-ordered by the number of survival- or survival+ sites per gene.
(XLSX)
