# Supplementary figures and images for: Pancreatic Cancer Patient Survival Correlates with DNA Methylation of Pancreas Development Genes
Source: PLoS One. 2015 Jun 3;10(6):e0128814. doi: 10.1371/journal.pone.0128814 (PMC4454596; doi:10.1371/journal.pone.0128814)

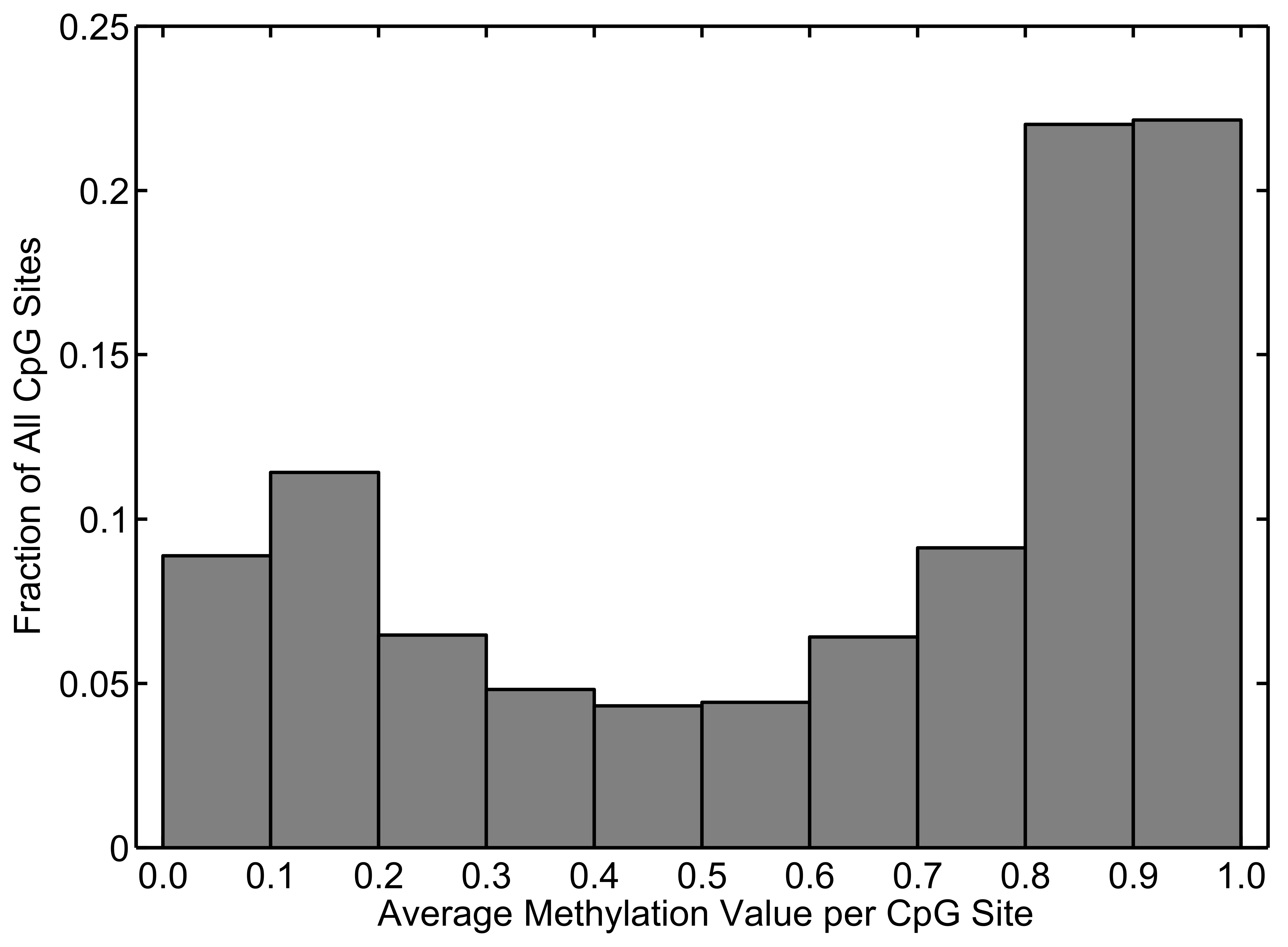

Supplement: S1 Fig — (TIFF) [file pone.0128814.s001.tiff]

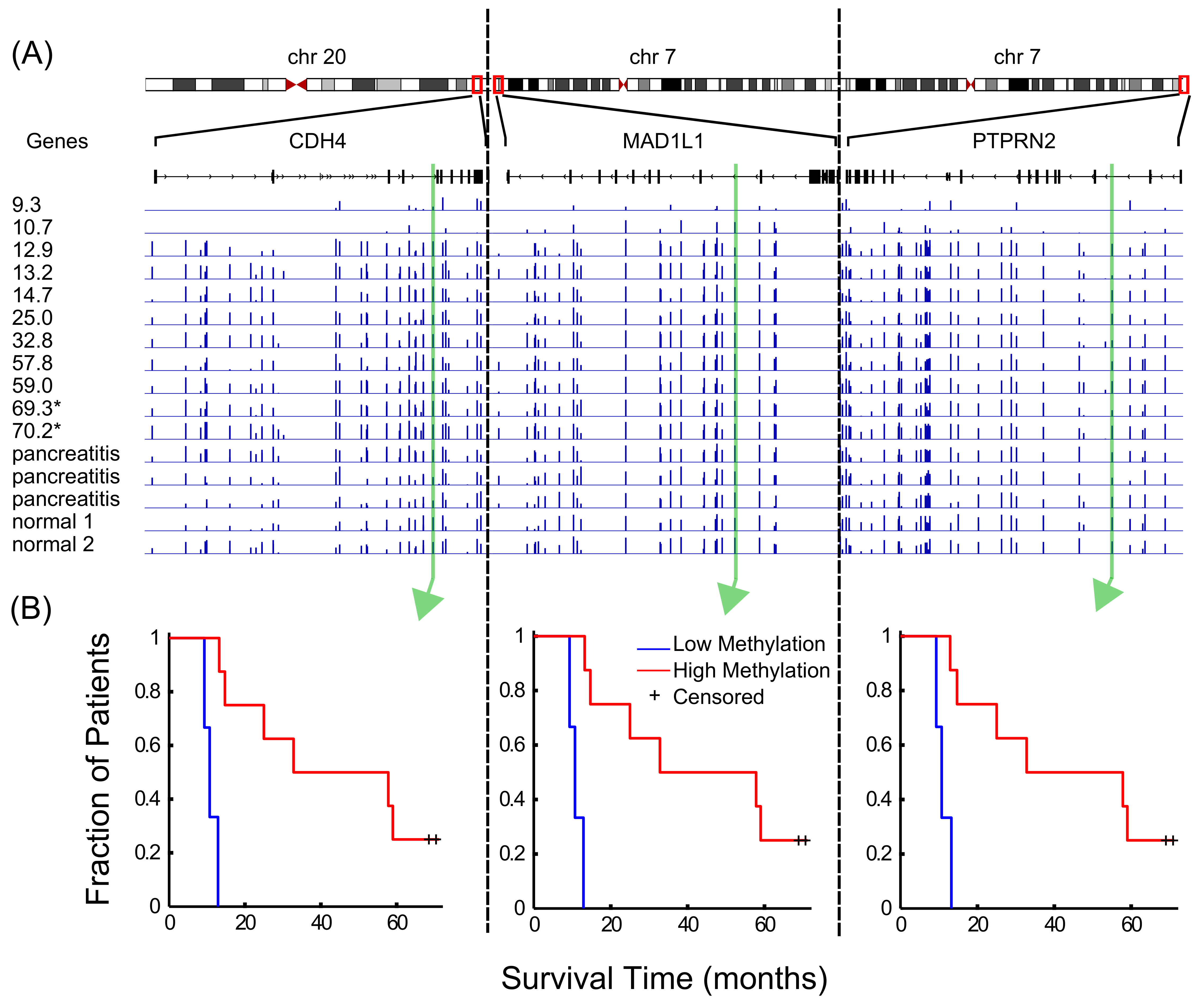

Supplement: S2 Fig — (TIFF) [file pone.0128814.s002.tiff]
